# Supplementary material for: Effects of pain education on disability, pain, quality of life, and self-efficacy in chronic low back pain: A randomized controlled trial
Source: PLoS One. 2024 May 28;19(5):e0294302. doi: 10.1371/journal.pone.0294302 (PMC11132453; doi:10.1371/journal.pone.0294302)
Supplement: S1 File — (DOCX) [file pone.0294302.s001.docx]

**Schedule of enrolment, Intervention, and assessment, the SPIRIT guidelines for pain education intervention RCT among chronic low back pain**

| **Study period** | **Screening** | **Baseline (T0)** | **Training Sessions** | **Intervention Period in weeks** | | | | | | **Post interventions Assessment (T1)** |
| --- | --- | --- | --- | --- | --- | --- | --- | --- | --- | --- |
| **Time (Weeks)** | **-4 to -1** |  | **S1 to S6** | **1** | **2** | **3** | **4** | **5** | **6** |  |
| Patient Data |  | **x** |  |  |  |  |  |  |  |  |
| Informed Consent |  | **x** |  |  |  |  |  |  |  |  |
| Demographic Data |  | **x** |  |  |  |  |  |  |  |  |
| Medical History |  | **x** |  |  |  |  |  |  |  |  |
| Allocation |  | **x** |  |  |  |  |  |  |  |  |
| Control group |  |  | **x** | **x** | **x** | **x** | **x** | **x** | **x** | **x** |
| Intervention group |  |  | **x** | **x** | **x** | **x** | **x** | **x** | **x** | **x** |
| VAS |  | **x** |  |  |  |  |  |  |  | **x** |
| RMDQ |  | **x** |  |  |  |  |  |  |  | **x** |
| WHO5 INDEX |  | **x** |  |  |  |  |  |  |  | **x** |
| GSF |  | **x** |  |  |  |  |  |  |  | **x** |
| Compliance and motivation |  | **x** |  | **x** | **x** | **x** | **x** | **x** | **x** |  |
| Patient Diary |  |  | **x** | **x** | **x** | **x** | **x** | **x** | **x** |  |
| Routine PT |  |  |  | **x** | **x** | **x** | **x** | **x** | **x** |  |
| Adverse Effects |  |  |  | **x** | **x** | **x** | **x** | **x** | **x** |  |
