# Supplementary material for: Effects of pain education on disability, pain, quality of life, and self-efficacy in chronic low back pain: A randomized controlled trial
Source: PLoS One. 2024 May 28;19(5):e0294302. doi: 10.1371/journal.pone.0294302 (PMC11132453; doi:10.1371/journal.pone.0294302)
Supplement: S2 File — (PDF) [file pone.0294302.s002.pdf]

“KNOW YOUR PAIN”

(PAIN EDUCATION MANUAL version 1.0)

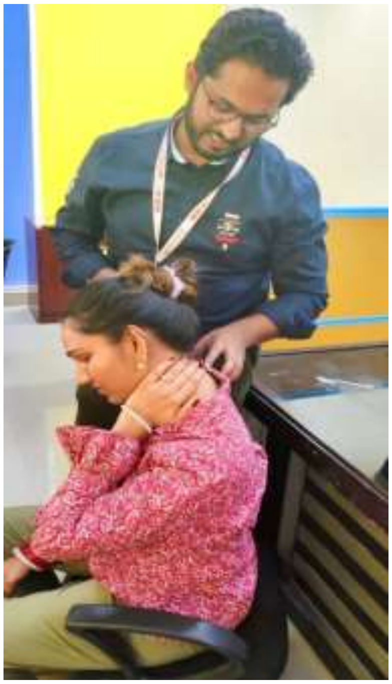

Prepared by: Mohammad Sidiq

Guide: Professor Arunachalam Ramachandran

Madhav University Abu Road Rajasthan

Pain is an emotional experience, tissue injury is not necessary" IASP

Table of Contents

PAIN EDUCATION MANUAL

Cover Page 1

Acknowledgement 3

Preface 4

Overview 5

CHAPTER 1 6

Chapter 2 7

Chapter 3 8

Chapter 4 9

Pain education MCQs 10

Future Recommendations 11

Flash Cards ....

References 12

Acknowledgement

I would like to take this opportunity to thank and express my sincere gratitude to the personalities in Physiotherapy research like David Butler, Louis Gifford, Lorimer Moseley, Michael Thacker, Andrew Louw, and Prashant Mukkanavar have been urging physiotherapists to adopt a new paradigm for understanding pain for more than twenty years. They may have been motivated by Patrick Wall's intellectual bravery. When we take into account circumstances where pain is felt even in the absence of tissue damage or when a person does not feel pain despite obvious tissue damage, the tissue-injury paradigm is rendered obsolete. The vast majority of clinical presentations of individuals with pain and 21st century pain neuroscience support a paradigm that emphasizes brain anatomy and function. This paradigm admits that nociception related to tissue damage is adjustable at the periphery, at the spinal cord, and in the brain, but it does not dismiss tissue-based pathology. Rather recommend a bio-psychosocial.

Overview

Background

Pain education varies from conventional educational approaches like back-schooling and biomechanical models. By avoiding anatomical or biomechanical models in favor of neurophysiology, neurobiology, and the processing and representation of pain(Brox et al., 2008). The best way to define pain education is as a lesson or series of lessons explaining the neurobiology, neurophysiology, and nervous system's role in processing pain. Instead of using the conventional model of connective tissue damage or nociception, pain education aims to explain how the nervous system interprets information from the tissues through synaptic activity, peripheral nerve sensitization, central sensitization, and brain processing, and that neural activation, as either upregulation or downregulation, has the capacity to modulate pain experience. So, patients are made aware of the fact that pain is not always an accurate indicator of the condition of the tissues and that pain is instead a result of the nervous system's processing of the injury in conjunction with numerous psycho-social factors(Louw et al., 2011)

Preface:

The field of pain management emphasizes the role of a pain education intervention for patients with a broad spectrum of conditions. The pioneers had called for establishment of the pain education guide and content representing regional social construct. This first edition of the pain education manual is a good beginning for developing a future comprehensive and effective educational intervention tool. The author has developed this manual as preliminary work, and I am sure that the team will develop a more refined version with additional content to improve pain management. I hope that you will find the contents and interventions contained herein of good value in alleviating pain and thereby improving the outcome of your rehabilitation service.

Regards

Professor Dr Balamurugan Janakiraman

Time Requirements (30 minutes)

First Session:

Preparation (Receiving the patient in a warm way).

The therapists or clinicians should developing a rapport for building trust with the patients

Materials required: Chair, Table, and Couch (for patients who want to lie down or have more pain while sitting), Pain education materials (Flash cards, AV aids,

Mode of Delivery: Face to Face interaction across the table, Handing over flash cards.

In the First Session, after receiving the patients, an **i**nformed consent is signed. Then a thorough physical examination is done and history taking. No pain education is given in the first session, but they are asked to come back but they are informed that this will take another 3 sessions.

Chapter 2

Time Requirements (30 minutes)

In the second session, receiving the patient, asking questions like "How do you feel today", recording their pain since first session. Even if any patient tells he/she doesn't have pain today, we need to educate them to complete all the 4 sessions of pain education course to avoid any recurrence of pain and for better understanding of pain . Now asking patients about the knowledge of pain and source of knowledge, myths, wrong misconceptions, De-educate starts (including correcting any misconceptions, myths and false information is corrected. Impart correct information.

Time Requirements (30 minutes)

In the third session, Revise, recap about the last pain education session actually learning retention. Here flash cards are introduced regarding and neurophysiology and neurobiology of pain are explained. Patient are asked to e go home, think and come back and write down anything regarding their pain experiences. This concludes the third session.

C

hapter 4

Time Requirements (30 minutes)

In the Last session, Revise, recap about all the three sessions. Patients are asked about any questions they have and diary is checked if they have written anything regarding their pain experiences. In the final session of PE few questions are asked regarding the concept of pain, central sensitization to check the retention and any learning they got during the course.

Pain MCQs

**1.**	**What is the duration of the pain required to be termed chronic?**
Options	I, 1 months
	II, 2 months
	III, More than 3 Months
**2.**	**What causes central sensitization?**
Options	I, Due to hypersensitivity of the peripheral nerves
	II, Due to hypersensitivity to movements
	III, Due to hypersensitivity of brain to normal sensations and repeated painful nerve stimulation
**3.**	**What is Kinesiophobia?**
Options	I, Any movement causes pain
	II, Normal movements exaggerate pain experiences
	III, Fear related pain experienced in chronic pain patients
**4.**	**What is pain education?**
Options	I, It is exercise induced education
	II, It is a strategy to reconceptualize the pain perception and knowledge
	III, It is a cognitive psychological therapy
**5.**	**How many sessions are required for pain education?**
Options	I, One
	II, two
	III, Three
	IV, More than five.

Flash Cards

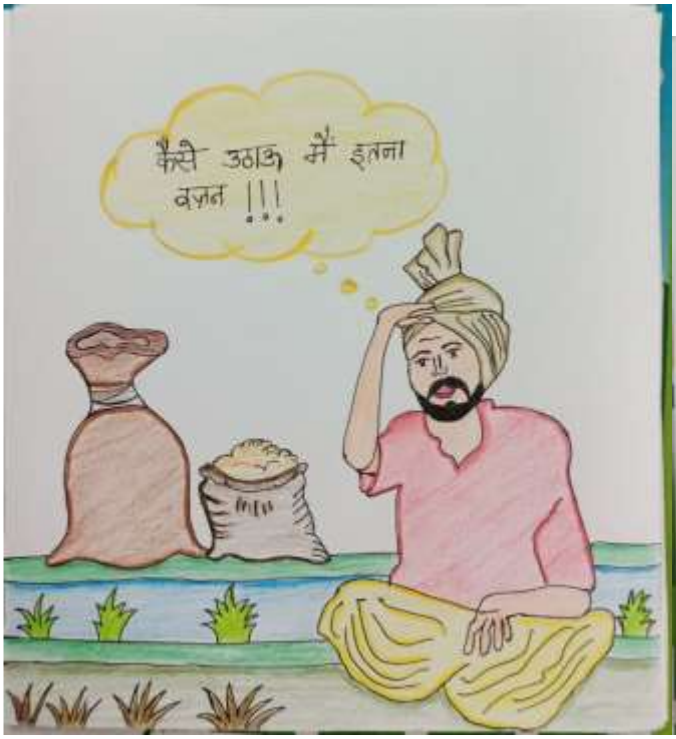
कैसे उठाऊ मैं इतना वज़न !!!

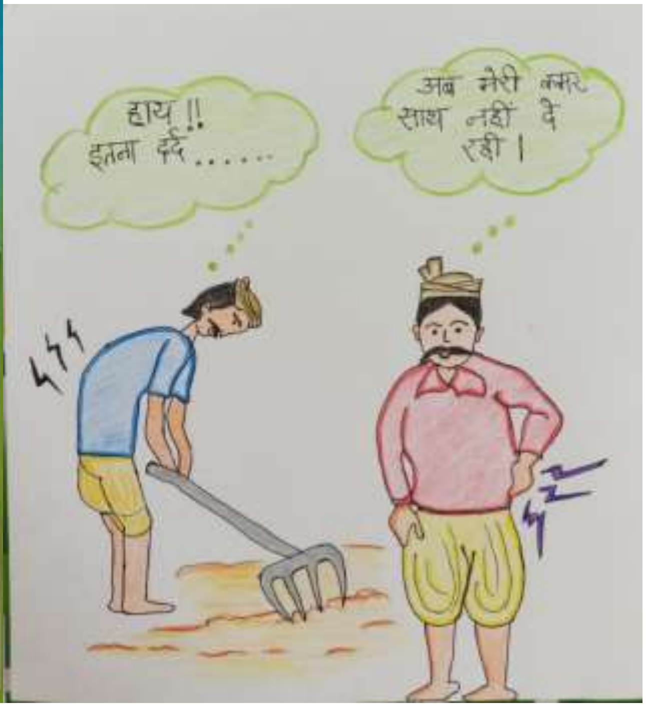
हाय !!
इतना दर्द......
अब मेरी कमर साथ नहीं दे रही।

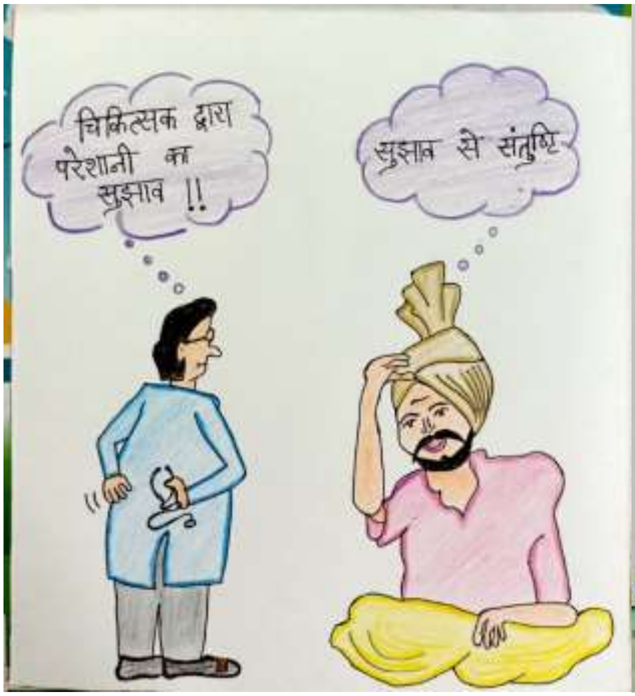
चिकित्सक द्वारा परेशानी का सुझाव !!
सुझाव से संतुष्टि

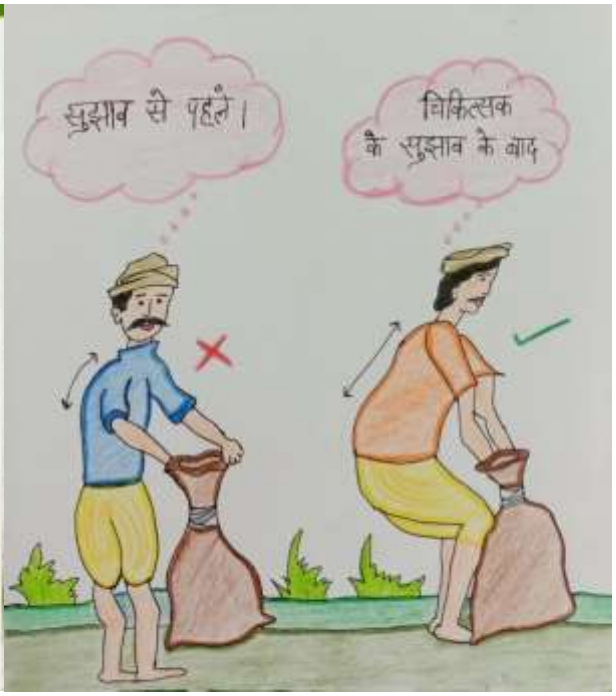
सुझाव से पहले।
चिकित्सक के सुझाव के बाद

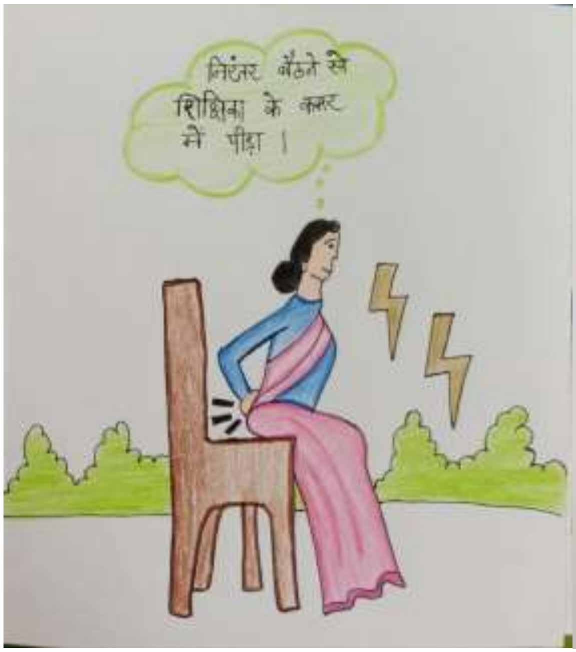
निरंतर बैठने से शिक्षिका के कमर में पीड़ा ।

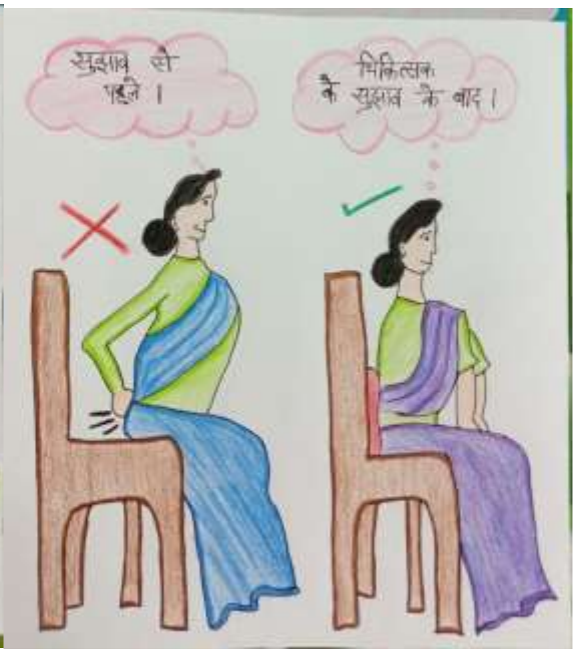
सुझाव से पहले ।
चिकित्सक के सुझाव के बाद ।

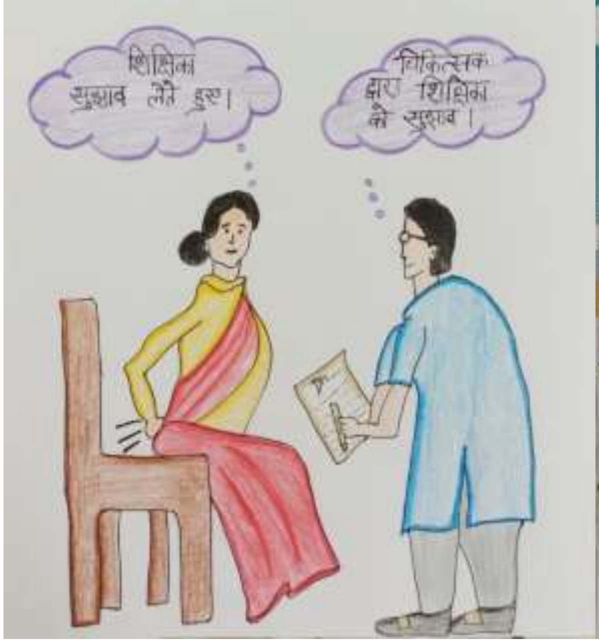
शिक्षिका सुझाव लेते हुए।
चिकित्सक द्वारा शिक्षिका को सुझाव ।

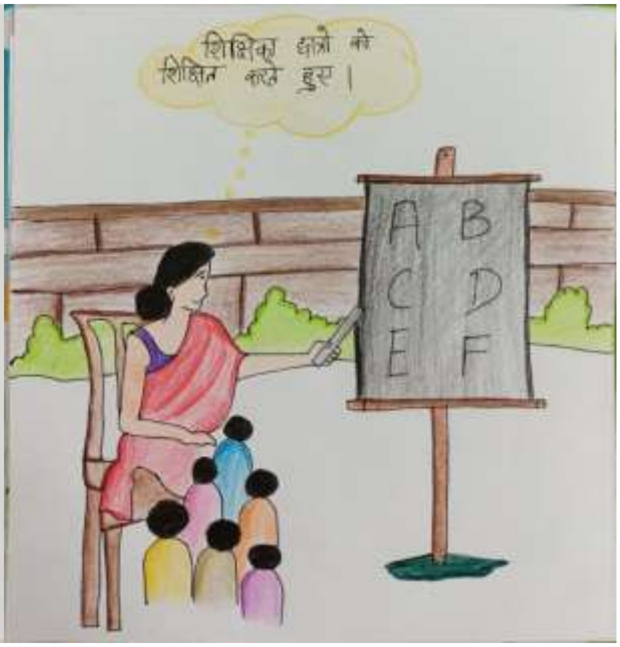
शिक्षिका छात्रों को शिक्षित करते हुए ।
A B
C D
E F

References:

Brox, J. I., Storheim, K., Grotle, M., Tveito, T. H., Indahl, A., & Eriksen, H. R. (2008). Systematic review of back schools, brief education, and fear-avoidance training for chronic low back pain. *Spine Journal*, *8*(6), 948–958. https://doi.org/10.1016/j.spinee.2007.07.389

Louw, A., Diener, I., Butler, D. S., Puentedura, E. J. (2011). The effect of neuroscience education on pain, disability, anxiety, and stress in chronic musculoskeletal pain. *Archives of Physical Medicine and Rehabilitation*, *92*(12), 2041–2056. https://doi.org/10.1016/j.apmr.2011.07.198

Jones, Caitlin M P et al. 2021. "Advice and Education Provide Small Short-Term Improvements in Painand Disability in People with Non-Speci Fi c Spinal Pain : A Systematic Review."

Louw, Adriaan, Emilio J Puentedura, Ina Diener, et al. 2016. "Pain Neuroscience Education : Which Pain Neuroscience Education Metaphor Worked Best ?" : 1–7.

Louw, Adriaan, Ina Diener, Merrill R Landers, Kory Zimney, et al. 2016. "Three-Year Follow-up of a Randomized Controlled Trial Comparing Preoperative Neuroscience Education for Patients Undergoing Surgery for Lumbar Radiculopathy." 2(4): 289–98.

Louw, Adriaan, Kory Zimney, Emilio J. Puentedura, and Ina Diener. 2016. "The Efficacy of Pain Neuroscience Education on Musculoskeletal Pain: A Systematic Review of the Literature." *Physiotherapy Theory and Practice* 32(5): 332–55.

Moseley, G L. 2003. "A Pain Neuromatrix Approach to Patients with Chronic Pain." 8: 130–40.

Moseley, G Lorimer. 2003. "Joining Forces – Combining Cognition-Targeted Motor Control Training withGroup or Individual Pain Physiology Education : A Successful Treatment For Chronic Low Back Pain ." 11(2): 88–94.

Moseley, G Lorimer, and David S Butler. 2015. "15 Years of Explaining Pain - The Past, Present andFuture." *Journal of Pain*. http://dx.doi.org/10.1016/j.jpain.2015.05.005.

Moseley, G Lorimer, Michael K Nicholas, and Paul W Hodges. 2004. "O RIGINAL A RTICLE A Randomized Controlled Trial of Intensive Neurophysiology Education in Chronic Low Back Pain." 20(5): 324–30.

Moseley, Lorimer. 2002. "Combined Physiotherapy and Education Is Efficacious for Chronic Low BackPain." *Australian Journal of Physiotherapy* 48(4): 297–302. http://dx.doi.org/10.1016/S0004-9514(14)60169-0. 2003.

"Unraveling the Barriers to Reconceptualization of the Problem in Chronic Pain: The Actual and Perceived Ability of Patients and Health Professionals to Understand the Neurophysiology." *Journal ofPain* 4(4): 184–89.

"अपना दर्द जानो"

दर्द शिक्षा मैनुअल

द्वारा तैयार: मोहम्मद सिद्दीक

पीएचडी विद्वान

मार्गदर्शक: प्रोफेसर अरुणाचलम रामचंद्रन

माधव विश्वविद्यालय आबू रोड राजस्थान

Prepared by
Prof Mohammad Sidiq

विषयसूची

दर्द शिक्षा मैनुअल

कवर पेज 1

पावती 3

प्रस्तावना 4

सिंहावलोकन 5

अध्याय 1 5

अध्याय 2 6

अध्याय 3 7

अध्याय 4 8

दर्द शिक्षा एमसीक्यू 9-10

सन्दर्भ 11

स्वीकृति

दर्द एक भावनात्मक अनुभव है, ऊतक की चोट जरूरी नहीं है' आईएएसपी

मैं डेविड बटलर, लुइस गिफोर्ड, लोरिमर मोसले, माइकल थैकर, एंड्रयू लूव और प्रशांत मुक्कनवर जैसे भौतिक चिकित्सा अनुसंधान में व्यक्तित्वों को धन्यवाद देने और अपनी कृतज्ञता व्यक्त करने के लिए इस अवसर को लेना चाहता हूं, फिजियोथेरेपिस्टों से समझने के लिए एक नया प्रतिमान अपनाने का आग्रह कर रहे हैं। बीस से अधिक वर्षों के लिए दर्द। वे पैट्रिक वॉल की बौद्धिक बहादुरी से प्रेरित हो सकते हैं। जब हम उन परिस्थितियों को ध्यान में रखते हैं जहां ऊतक के अभाव में भी दर्द महसूस होता है

प्रस्तावना

दर्द प्रबंधन का क्षेत्र परिस्थितियों के व्यापक स्पेक्ट्रम वाले मरीजों के लिए दर्द शिक्षा हस्तक्षेप की भूमिका पर जोर देता है। अग्रदूतों ने दर्द शिक्षा गाइड और क्षेत्रीय सामाजिक निर्माण का प्रतिनिधित्व करने वाली सामग्री की स्थापना का आह्वान किया था। दर्द शिक्षा मैनुअल का

यह पहला संस्करण भविष्य में व्यापक और प्रभावी शैक्षिक हस्तक्षेप उपकरण विकसित करने के लिए एक अच्छी शुरुआत है। लेखक ने इस मैनुअल को प्रारंभिक कार्य के रूप में विकसित किया है, और मुझे यकीन है कि टीम दर्द प्रबंधन में सुधार के लिए अतिरिक्त सामग्री के साथ एक अधिक परिष्कृत संस्करण विकसित करेगी।

मुझे आशा है कि आपको इसमें निहित सामग्री और हस्तक्षेप दर्द को कम करने और इस तरह आपकी पुनर्वास सेवा के परिणाम में सुधार

करने के लिए अच्छे मूल्य के मिलेंगे।

सम्मान

प्रोफेसर डॉ बालमुरुगन जानकीरमन

अध्याय 1

समय की आवश्यकताएं (30 मिनट)

पहला सत्र: तैयारी (रोगी का गर्मजोशी से स्वागत करना)। चिकित्सक या चिकित्सकों को रोगियों के साथ विश्वास बनाने के लिए एक तालमेल विकसित करना चाहिए। बैठे।

दर्द शिक्षा सामग्री (फ्लैश कार्ड, एवी एड्स)

डिलीवरी का तरीका: टेबल पर आमने-सामने बातचीत, फ्लैश कार्ड सौंपना।

पहले सत्र में, रोगियों को प्राप्त करने के बाद, ए सूचित सहमति पर हस्ताक्षर किए हैं। फिर पूरी तरह से शारीरि परीक्षा हो चुकी है और इतिहास लिया जा रहा है। पहले सत्र में कोई दर्द शिक्षा नहीं दी जाती है, लेकिन उन्हें वापस आने के लिए कहा जाता है लेकिन उन्हें सूचित किया जाता है कि इसमें 3 सत्र और लगेंगे।

अध्याय दो

समय की आवश्यकताएं (30 मिनट)

दूसरे सत्र में, रोगी को प्राप्त करना, पूछना "आज आप कैसा महसूस कर रहे हैं" जैसे प्रश्नों को रिकॉर्ड कर रहे हैं पहले सत्र से दर्द। भले ही कोई मरीज उसे बताए आज दर्द नहीं है, हमें उन्हें शिक्षित करने की जरूरत है दर्द शिक्षा पाठ्यक्रम के सभी 4 सत्रों को पूरा करें दर्द की पुनरावृत्ति से बचें और बेहतर समझ के लिए दर्द की । अब मरीजों से दर्द के ज्ञान के बारे में पूछ रहे हैं और ज्ञान, मिथकों, गलत भ्रांतियों का स्रोत। डी-एजुकेट शुरू होता है (किसी भी गलत धारणा, मिथक और गलत जानकारी को सही करने सहित) को सही किया जाता है। सही जानकारी दें।

अध्याय 3

समय की आवश्यकताएं (30 मिनट)

तीसरे सत्र में, पिछले दर्द शिक्षा सत्र वास्तव में सीखने की अवधारण के बारे में पुनरीक्षण करें। यहां फ्लैश कार्ड के बारे में बताया गया है और दर्द के न्यूरोफिजियोलॉजी और न्यूरोबायोलॉजी के बारे में बताया गया है। रोगी को ई घर जाने, सोचने और वापस आने और कुछ भी लिखने के लिए कहा जाता है

अध्याय 4

समय की आवश्यकताएं (30 मिनट)

अंतिम सत्र में, तीनों के बारे में पुनरीक्षण, पुनर्कथन करें सत्र। मरीजों से उनके किसी भी सवाल के बारे में पूछा जाता है, और डायरी की जाँच की जाती है कि क्या उन्होंने कुछ लिखा है उनके दर्द के अनुभवों के बारे में। के अंतिम सत्र में पीई की अवधारणा के संबंध में कुछ प्रश्न पूछे जाते हैं दर्द, केंद्रीय संवेदीकरण अवधारण और किसी की जाँच करने के लिए पाठ्यक्रम के दौरान उन्हें सीखने को मिला।

बहु विकल्पीय प्रश्न

1. **जीर्ण होने के लिए आवश्यक दर्द की अवधि क्या है?**

विकल्प

I, 1 महीना

II, द्वितीय, 2 महीने

III, 3 महीने से अधिक

2. **केंद्रीय संवेदीकरण का क्या कारण बनता है?**

विकल्प

I, परिधीय तंत्रिका की अतिसंवेदनशीलता के कारण

II, गतिविधियों के प्रति अतिसंवेदनशीलता के कारण

III, सामान्य संवेदनाओं के प्रति मस्तिष्क की अतिसंवेदनशीलता और बार-बार दर्दनाक तंत्रिका उत्तेजना के कारण

3.

विकल्प

किनेसियोफोबिया क्या है?

I, कोई भी हरकत दर्द का कारण बनती है

II, सामान्य गतिविधियां दर्द के अनुभवों को बढ़ा देती हैं

III, पुराने दर्द के रोगियों में भय संबंधी दर्द का अनुभव

4.

विकल्प

दर्द शिक्षा क्या है?

I, यह व्यायाम प्रेरित शिक्षा है

II, यह दर्द की धारणा और ज्ञान को फिर से अवधारणा बनाने

की रणनीति है

III, यह एक संज्ञानात्मक मनोवैज्ञानिक चिकित्सा है

**5.**	**दर्द शिक्षा के लिए कितने सत्र आवश्यक हैं?**
**विकल्प**	मैं, एक
	द्वितीय, दो
	तृतीय, तीन
	चतुर्थ, पांच से अधिक।

Flash card 1

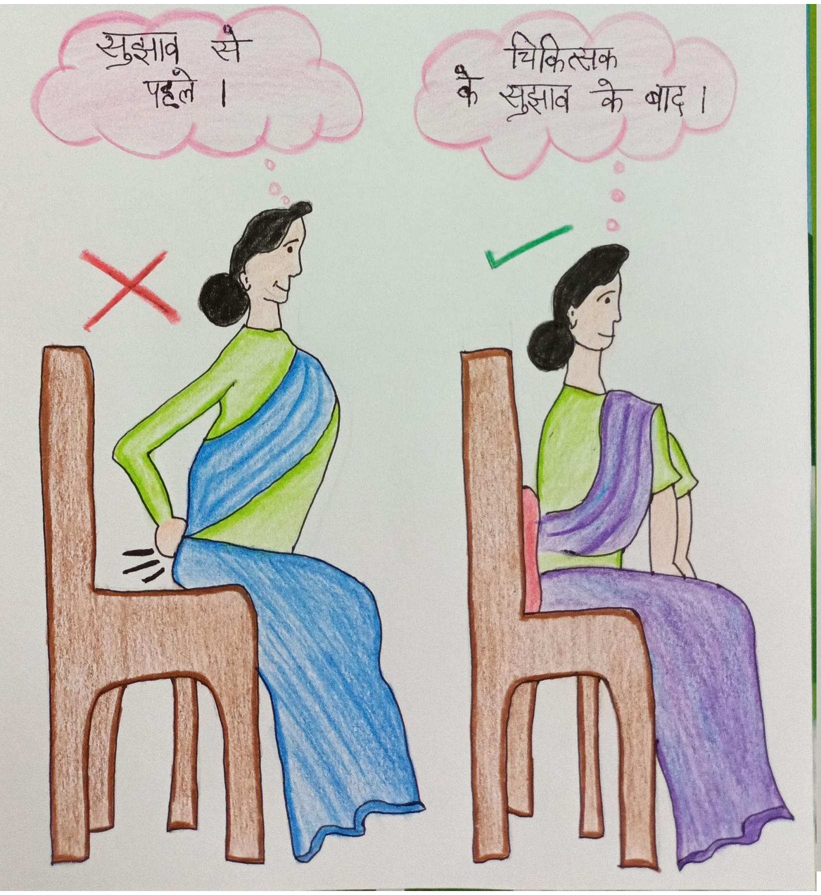

Flash card 2

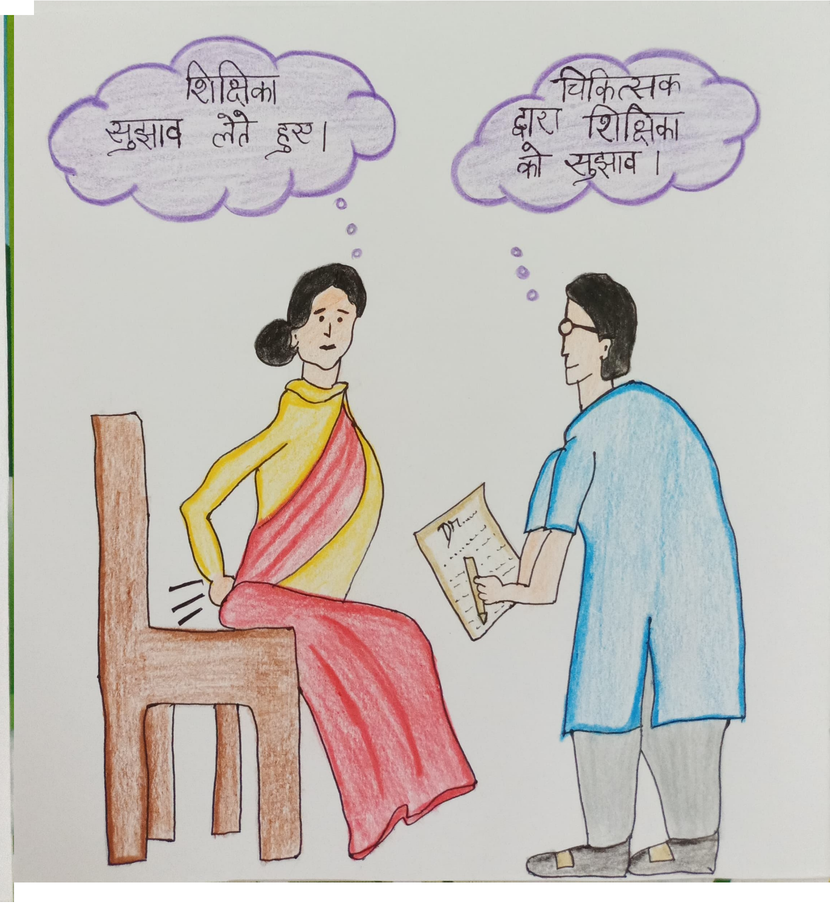

Flash card 3

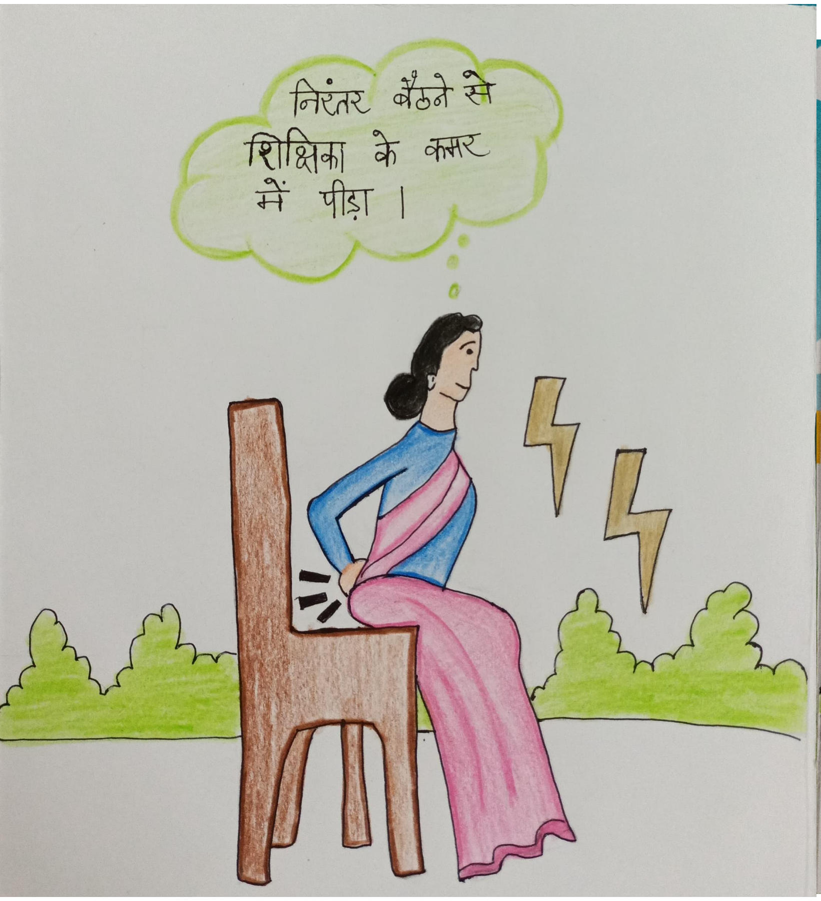

Flash card 4

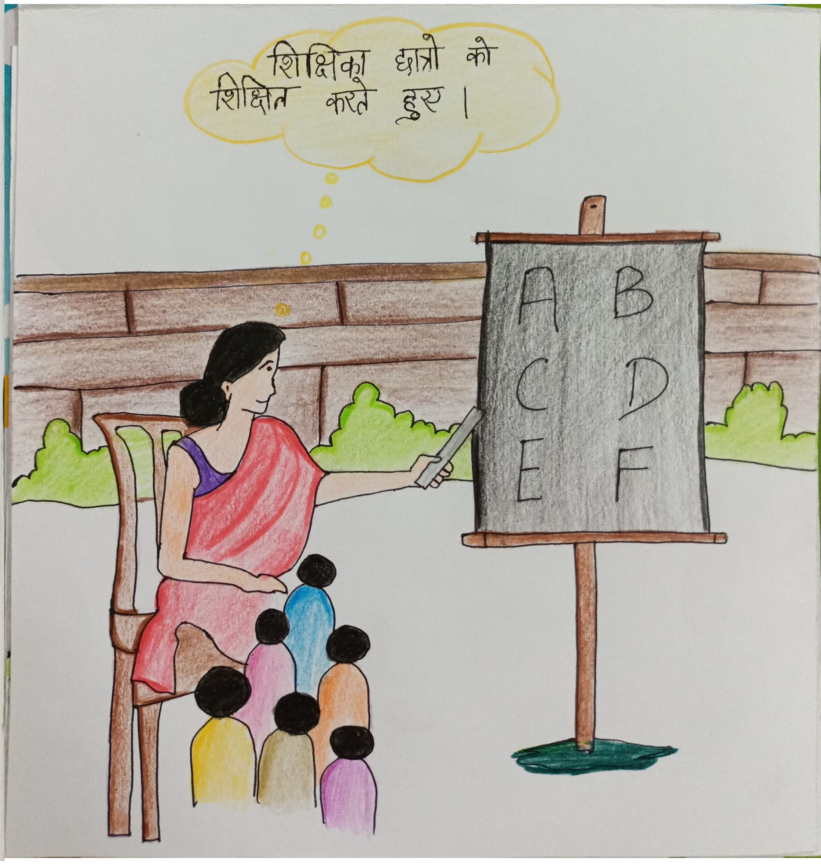

Flash card 5

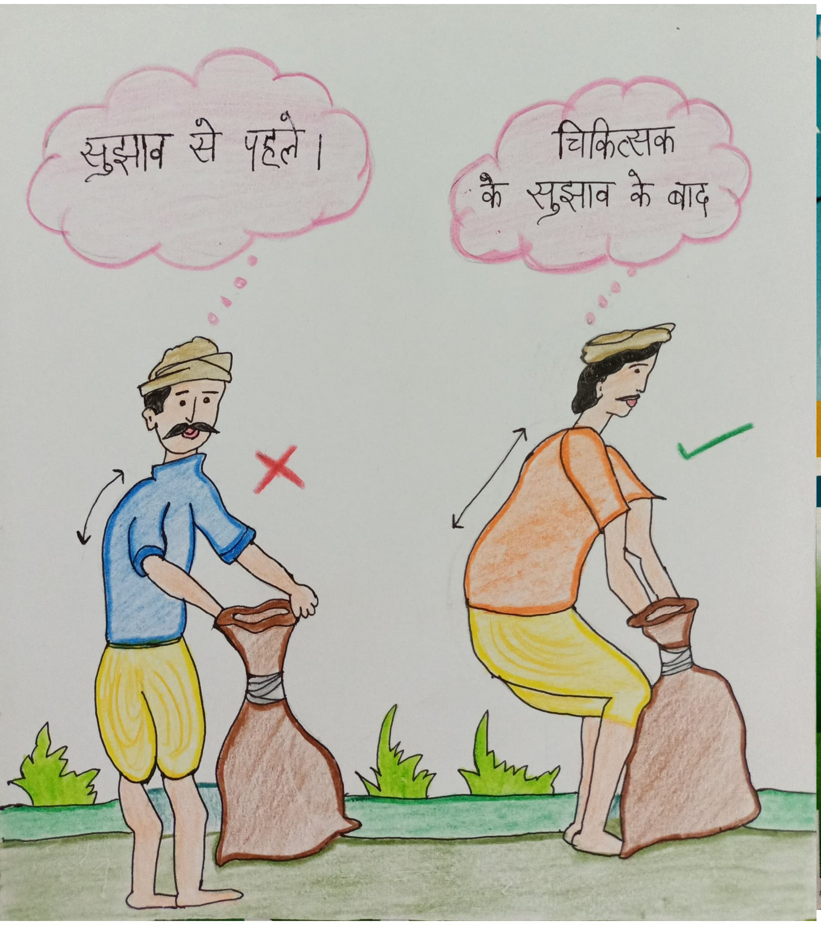

Flash card 6

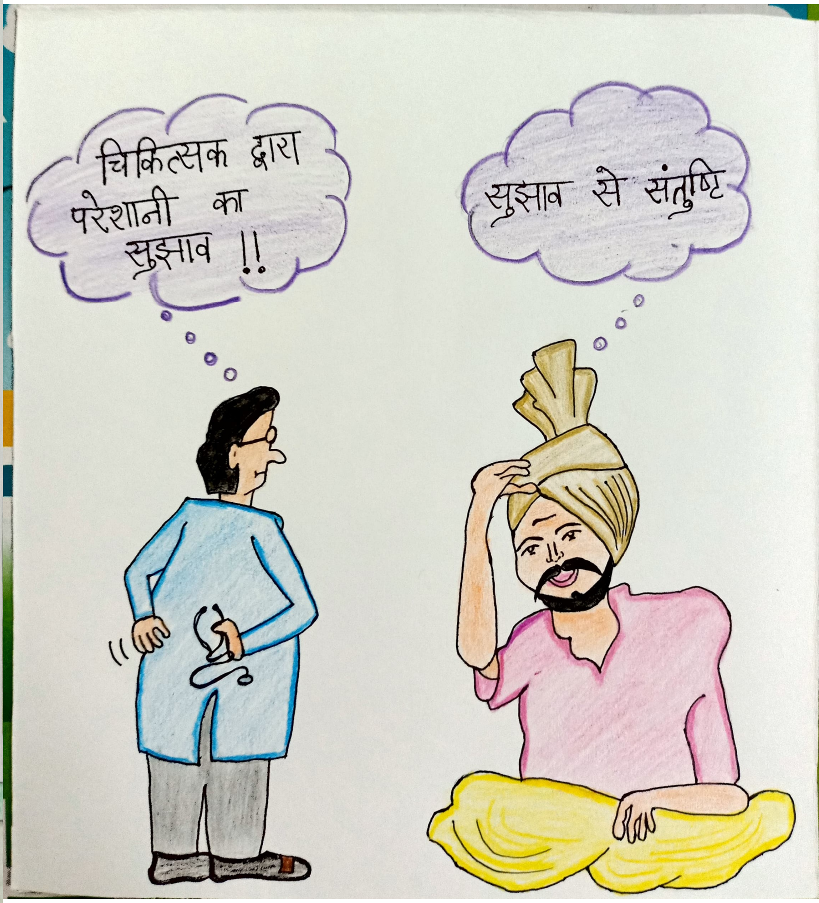

Flash card 7

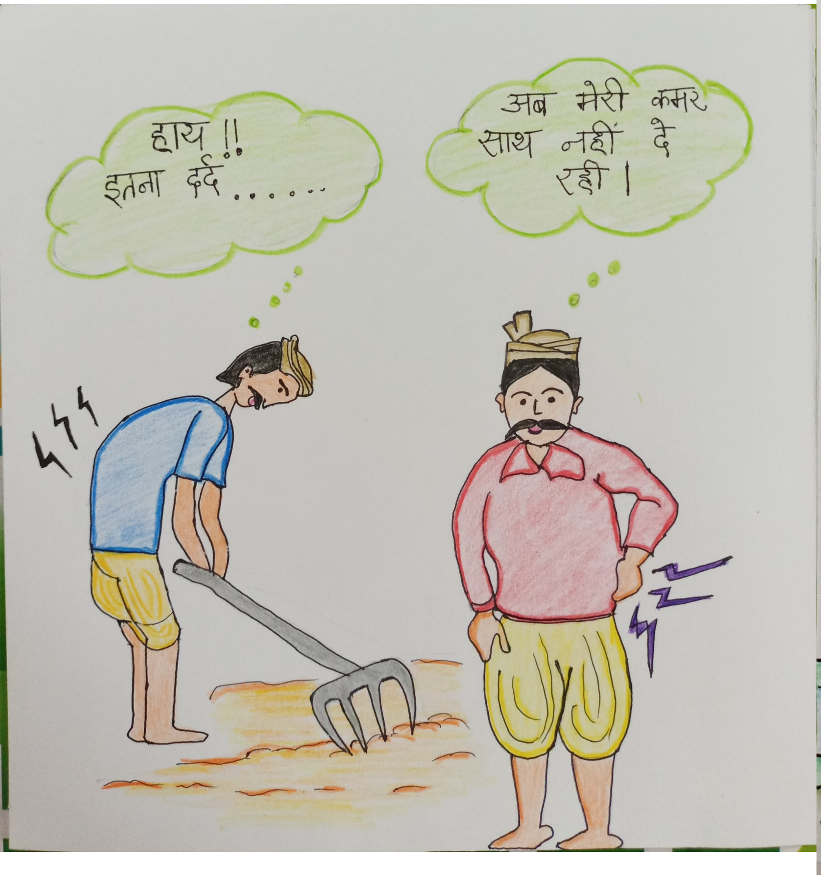

Flash card 8

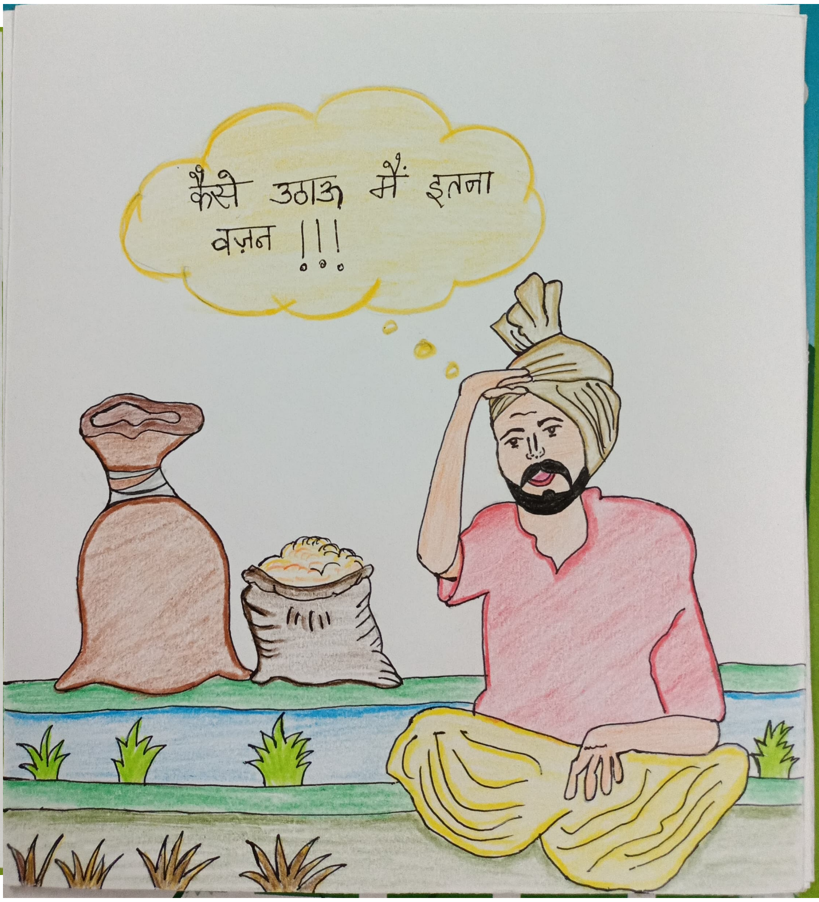

संदर्भ:

Brox, J I et al. 2008. “Systematic Review of Back Schools , Brief Education , and Fear-Avoidance Training for Chronic Low Back Pain.” *The Spine Journal* 8(6): 948–58. http://dx.doi.org/10.1016/j.spinee.2007.07.389.

Louw, Adriaan, Ina Diener, David S. Butler, and Emilio J. Puentedura. 2011. “The Effect of Neuroscience Education on Pain, Disability, Anxiety, and Stress in Chronic Musculoskeletal Pain.” *Archives of Physical Medicine and Rehabilitation* 92(12): 2041–56. http://dx.doi.org/10.1016/j.apmr.2011.07.198.

Jones, Caitlin M P et al. 2021. “Advice and Education Provide Small Short-Term Improvements in Pain and Disability in People with Non-Speci Fi c Spinal Pain : A Systematic Review.”

Louw, Adriaan, Emilio J Puentedura, Ina Diener, et al. 2016. “Pain Neuroscience Education : Which Pain Neuroscience Education Metaphor Worked Best ?” : 1–7.

Louw, Adriaan, Ina Diener, Merrill R Landers, Kory Zimney, et al. 2016. “Three-Year Follow-up of a Randomized Controlled Trial Comparing Preoperative Neuroscience Education for Patients Undergoing Surgery for Lumbar Radiculopathy.” 2(4): 289–98.

Louw, Adriaan, Kory Zimney, Emilio J. Puentedura, and Ina Diener. 2016. “The Efficacy of Pain Neuroscience Education on Musculoskeletal Pain: A Systematic Review of the Literature.” *Physiotherapy Theory and Practice* 32(5): 332–55.

Moseley, G L. 2003. “A Pain Neuromatrix Approach to Patients with Chronic Pain.” 8: 130–40.

Moseley, G Lorimer. 2003. “Joining Forces – Combining Cognition-Targeted Motor Control Training with Group or Individual Pain Physiology Education : A Successful Treatment For Chronic Low Back Pain .” 11(2): 88–94.

Moseley, G Lorimer, and David S Butler. 2015. “15 Years of Explaining Pain - The Past, Present and Future.” *Journal of Pain*. http://dx.doi.org/10.1016/j.jpain.2015.05.005.

Moseley, G Lorimer, Michael K Nicholas, and Paul W Hodges. 2004. “O RIGINAL A RTICLE A Randomized Controlled Trial of Intensive Neurophysiology Education in Chronic Low Back Pain.” 20(5): 324–30.

Moseley, Lorimer. 2002. “Combined Physiotherapy and Education Is Efficacious for Chronic Low Back Pain.” *Australian Journal of Physiotherapy* 48(4): 297–302. http://dx.doi.org/10.1016/S0004-9514(14)60169-0. 2003.

“Unraveling the Barriers to Reconceptualization of the Problem in Chronic Pain: The Actual and Perceived Ability of Patients and Health Professionals to Understand the Neurophysiology.” *Journal of Pain* 4(4): 184–89.
