# Supplementary material for: Effects of pain education on disability, pain, quality of life, and self-efficacy in chronic low back pain: A randomized controlled trial
Source: PLoS One. 2024 May 28;19(5):e0294302. doi: 10.1371/journal.pone.0294302 (PMC11132453; doi:10.1371/journal.pone.0294302)
Supplement: Attachment — Submitted filename: Authors response letter.docx [file pone.0294302.s004.docx]

**Jan 2024**

PLOS ONE

Manuscript ID **PONE-D-23-35122**

**Effectiveness of pain education on pain, disability, quality of life and self-efficacy in chronic low back pain: A randomized controlled trial**

**Point-by-point response letter**

Dear Editor,

We express our sincere appreciation to the editor for the time and interest in this study.

Dear reviewers,

"We sincerely appreciate and thank you for your valuable comments, suggestions, and interest in this research paper. The reviewers and editor have provided constructive feedback and posed legitimate questions, significantly enhancing the quality of the manuscript. We have incorporated the suggested corrections and amendments. Furthermore, spelling, grammar, and sentence structure errors have been rectified, aligning the manuscript with the journal's style requirements.

It's important to note that the reference line number for any modification corresponds to the track change manuscript, not the clean manuscript.

*All the authors agreed on the changes made in response to the reviewer's comments.*

*Please find our responses below:*

With regards

| **S no** | **Reviewers comment** | **Author’s response** |
| --- | --- | --- |
| **1.** | Change the title to: Effects of pain education on disability, pain, quality of life, and self-efficacy in chronic low back pain: A randomized controlled trial | Changed as recommended  *Page 01, lines 2 – 3* |
| **2** | Include keywords according to MeSH terms (https://www.ncbi.nlm.nih.gov/mesh/) | MeSH terms included in the manuscript as recommended.  *Page 03, line 65 -66, Abstract section> Keywords* |
| **3** | The objective stated in your abstract is different from the objective stated in your introduction. Please correct this. The sentence describing the objective must be exactly the same in both; | We have amended the changes. The objectives in abstract and in introduction are same now  *Page 2, Abstract section > background, line 42 -45.*  *Page 4, Introduction, line 115 -118*. |
| **4** | State a hypothesis in the introduction; | Hypothesis included in the introduction  *Page 4, Introduction, line 108 -111*. |
| **5** | The conclusion described in the abstract and the conclusion described after the discussion must be exactly the same; | Conclusion in the abstract and following discussion are same now.  *Page 2-3, Abstract section > conclusion, line 60-64.*  *Page 15, Conclusion section, line 408 -415.* |
| **6** | In the Methods (Data analysis) section, describe the classification used for the effect size (Cohen's d [https://pubmed.ncbi.nlm.nih.gov/37971135/]), since you reported it as n2 in Table 2 | A description of Cohen’s d is provided in data analysis section as recommended. And, change are made to table 2 accordingly.  *Methods section > Data analysis, page 8, lines 230 -233* |
| **7.** | In Table 1, report the body mass (kg) and stature (cm); | The variables are now included in *table 1, page 9* |
| **8.** | In Table 2, remove the description of within-group comparisons (not necessary for this RCT). Notice that your research question is answered by between-group comparisons | Removed as recommended, *Table 2 within group comparisons is deleted now. Page 11.* |
| **9** | In Table 3, change p = 0.000 to p <0.001; | Changes amended as recommended, *page 12.* |
| **10** | Submit the database with the data in unanalyzed values (i.e., absolute values) as a supplemental file (Excel and English) | Supplementary raw data included as supplementary file in the excel form. |
| **11** | Please ensure that your manuscript meets PLOS ONE's style requirements, including those for file naming. The PLOS ONE style templates can be found at <https://journals.plos.org/plosone/s/file?id=wjVg/PLOSOne_formatting_sample_main_b> ody.pdf and https://journals.plos.org/plosone/s/file?id=ba62/PLOSOne_formatting_sample_title_authors_affiliations.pdf. | Manuscripts now conforms to PLOS ONE style requirements. |
| **12** | In the online submission form, you indicated that [All the data supporting the findings of this trial is presented and the full dataset are available from the principal investigator on request.]. All PLOS journals now require all data underlying the findings described in their manuscript to be freely available to other researchers, either 1. In a public repository, 2. Within the manuscript itself, or 3. Uploaded as supplementary information | Uploaded as supplementary information |
| **13** | Please amend your authorship list in your manuscript file to include author Alagappan Thiyagarajan | Thank you for pointing out, this was wrongly entry in the Editorial submission webpage.  Authorship and author list is now updated in the Editorial webpage accordingly. |
| **14** | Please include captions for your Supporting Information files at the end of your manuscript, and update any in-text citations to match accordingly. Please see our Supporting Information guidelines for more information: http://journals.plos.org/plosone/s/supporting-information | Captions included at the end of the manuscript. |
| **15** | Reviewer # 1 |  |
| **16** | The study presents a solid scientific basis. There is significant thematic relevance and remarkable coherence in giving the context of previous literature, especially when considering the distinct cultural differences in each continent | Thanks for the encouraging comments |
| **17** | Main issues: MAJOR | |
| **18** | According to the journal's guidelines, including the trial or study's registration number in the abstract is essential | Trial registration included in the abstract. *Page 2, Abstract section > methods first para.* |
| **19** | The last paragraph of the introduction could be more concise, avoiding excessive repetition of terms | The last sentence in introduction is made concise with addition of hypothesis.  *Page 04, lines 109 – 117, last para of the introduction section*. |
| **20** | Improving the description of the standard physiotherapeutic treatment is essential. It would be relevant to provide details of the physical assessments carried out and clarify whether all patients received the same type of treatment regarding sets, repetitions and weights. This is especially crucial considering the diversity in the fitness levels of the participating patients. | All physiotherapy treatment was similar to all participants. The treatment was carried out based on a written protocol. Detailed description is provided in the method section for treatment to be replicated.  *Page 06, Methods section > Description of intervention, lines 164 -169.* |
| **21** | When writing the conclusion, it is important to exercise caution to ensure conciseness and a clear answer to the question raised in the introduction. The decision should be aligned with the results obtained, avoiding inappropriate wording | Inappropriate words removed and replaced with concise and clear terms. |
| **22** | Reviewer # 2 |  |
| **23** | This is an interesting study that is looking at the effect of pain education on pain intensity. However would benefit from some proof reading, as they are spelling mistakes in some places | Complete manuscript has being edited for grammar and punctuations. |
| **24** | Major observation: Primary outcomes measures are: The primary outcomes measures were related to the intensity of pain experienced during activities of daily life (ADL) and disability related to pain. Roland Morris Disability Questionnaire (RMDQ). The sample size is based on "mean score of pain intensity in chronic LBP patients (mean1 = 3.76, mean2 = 4.78, SD1 = 1.51, SD2 = + 1.91)". Its unclear whether this is a combination of of the two ADL and RMDQ – which by definition are co-primary endpoints. This needs to be reflected in the sample size, and the results as its confusing, there is reduction in disability and pain intensity. In addition, if the question was addressing two co-primary endpoints? Then more information is required in terms multiplicity testing or not and why? Or pre-specified interpretations in terms of whether intervention works if one or both or either outcomes are observed to be clinically significant. | The sample size calculation corrected as per the primary outcome.  *Page 06, Methods section > sample size calculation, lines 153 -156.* |
|  | Some further suggestions; |  |
| **25** | In the introduction section, mention that the primary was at 6 weeks With the primary outcome, can the authors include the collection time points, i.e. in the data analysis section, the authors mention, post intervention. Is this at 6 weeks, or at follow-up time point? | Thank you for pointing out – we have now included a mention at the *Methods > outcome measures*; a statement to clarify for the readers that the outcome measures were recorded at the baseline and at the end of 6 weeks (post-intervention), *Page 07, lines 196 – 197..* |
| **26** | As this is a randomised controlled trial, there needs to be distinction in terms of the analysis to reflect this. Its not clear what the main objective was | We have amended the changes. The objectives in abstract and in introduction are stated as recommended; *Page 2, Abstract section > background, line 42 -45. Page 4, Introduction, line 115 -118*. |
| **27** | Suggested to update the data analysis section, -To include that as this an RCT, this will be reported in accordance to the CONSORT guidelines? | The same is already stated in the methods section. Hence, please clarify if we need to state it again.  Methods section > page no 5, line 126. |
| **31** | Why was lost to follow-up not accounted for in the sample size, or include a sentence to justify why not, i.e. by design because the intervention last 6 weeks, so were the researchers confident enough that participants would adhere? | Yes, a contingency of 10% is customary. But, unfortunately we had feasibility constraints to include furthermore participants. We also had a priori plan of conducting a post-hoc analyses in case of drop-outs. |
| **32** | It is not recommended to test baseline characteristics, any differences between baseline characteristics would be due to chance. - see this interesting article  Correct Baseline Comparisons in a Randomized Trial Schober, Patrick MD, PhD, MMedStat*; Vetter, Thomas R. MD, MPH† "Traditionally, baseline balance is assessed by a series of hypothesis tests comparing each baseline variable between the groups, and a non-significant result (P > .05) has commonly been considered to indicate baseline balance. This approach is flawed.2 First, all patients are randomly sampled from the same population, and any differences must be due to chance. It thus does not make sense to test hypotheses of group differences at baseline. Second, hypothesis tests are greatly affected by sample size. In a smaller trial, marked differences between the groups can be non-significant, whereas in a larger trial, negligible differences could be significant." | We deeply appreciate you for enhancing our understanding about the statistical difference at the baseline.  We totally agree with the evidence you had cited here.  But, our concern is about some of the important non-clinical prognostic predictors of LBP prognosis that were non-proportionally distributed at the baseline between the groups and if these variables affect the prognosis.  That, prompted us to run a prognostic model. And, many researchers had recommended to verify these predictors & their effects on intervention?  Further, in line with your suggestion the statistical difference at baseline is not emphasized elsewhere in the manuscript.  Alessandra Narciso Garcia, Leonardo O. P. Costa, Luciola Da Cunha Menezes Costa, Mark Hancock & Chad Cook (2019): Do prognostic variables predict a set of outcomes for patients with chronic low back pain: a long-term follow-up secondary analysis of a randomized  control trial, Journal of Manual & Manipulative Therapy  Hayden JA, Chou R, Hogg-Johnson S, et al. Systematic reviews of low back pain prognosis had variable methods and results: guidance for future prognosis reviews. J Clin Epidemiol. 2009 Aug;62(8):781–796 e1. |
| **33** | This sentence is incomplete "Unadjusted mean (SD), and standard error (SE) of the mean difference (MD) between the groups with associated 95% confidence interval and p-value were computed for the outcome measures at baseline and post intervention"- using what statistical approach/model? | Thank you for notifying, we have now mentioned the model used accordingly. Data analysis section >>> page 08, lines 226 -227. |
| **34** | The authors jump straight to logistic prognostic model - which is not clear whether this was separate objective.  The most appropriate approach would be better to be clear as to whether the outcomes were analysed with the pre-specified baseline characteristics for assessing the treatment effect. The statistical analysis of prognostic model should be differentiated from the research question (primary) and secondary (which are questions assessing the effect of the intervention) - otherwise its confusing. | Yes, we agree with your comments. Based on the suggestions given we have now included statements in the background section highlighting the importance the factors that might predict the prognosis of CLBP – post interventions. |
| **35** | How was missing data handled- this is not mentioned? | Thank you, there were 3 instance of missing data at the baseline time point, we have now mentioned the statement regarding missing data handling.  *Page 09, results section, lines 258 -261.* |
| **36** | Also in the statistical analysis section, mention when the SAP was drafted etc | A statement of statistical analysis plan is now added. *Data analysis section > page 09, lines 242 – 244.* |
| **37** | How was adherence/compliance assessed | Treatment was delivered in person, and a study calendar was used to record patient adherence. A statement is now included. *Methods > Description of intervention, Page 07, lines 180-181.* |
| **38** | Table 1 - add percentage for gender (male and female) | Changes amended accordingly, page 9, table 01 |
| **39** | There is imbalance in terms of occupation, interestingly more housewives in the control group | Thank you for notifying, we have included a statement. *Page 9, Results section, baseline characteristics, lines 256-258.* |
| **40** | Additional information is missing in the randomisation section include allocation ratio | We have now included a statement on how we managed random allocation and the ratio of allocation.  *Methods > study design and ethical consideration, Page 5, lines 124-125.* |
| **41** | Figure 2 - put units on y-axis (e.g mean score difference | Changes amended accordingly |
| **42** | Discuss limitation in terms of generalizability, as this was in one single centre? | Thank you, we have include a statement now in the last para of discussion regarding limited external validity. |
| **43** | Reviewer # 3 |  |
| **44** | The manuscript is well-written and detailed, indicating a significant investment of time and effort. I will provide specific comments on the manuscript below | Thank you so much for the acknowledgement of our efforts, suggestions and comments. |
| **45** | Abstract: - I recommend revising the aim as follows: “This randomized controlled trial aimed to evaluate the effectiveness of pain education on pain levels, disability, quality of life, and self-efficacy in individuals with chronic low back pain (CLBP)” | Done accordingly, page2, abstract section, lines 44 -45 |
| **46** | Introduction: -It is crucial to include references to previous studies that have assessed the effect of PNE in patients with CLBP. Provide a summary of their findings and highlight the novelty of the present study.-The introduction should include the scientific background of the topic and an explanation of the rationale. -The authors need to clearly state the hypothesis of the study | Changes are amended accordingly in the introduction section  *Page 4, Introduction, line 108 -111*. |
| **47** | Methods: Reference “Figure 1” as the flow diagram in the manuscript.  If there were any modifications to the methods after trial registration, specify them. Provide a detailed description of the intervention called “standard physiotherapy." It would be important not only for transparency but also to offer valuable information for future studies. Assess and report on the blinding procedures. How can the authors ensure that appropriate blinding is achieved? Sample size: Recommend recalculating the sample size, especially considering the Roland-Morris Disability Questionnaire (RMDQ) as a primary outcome. | There were no modifications to methods after trial registration.  Detailed description is provided in the method section for treatment to be replicated.  *Page 06, Methods section > Description of intervention, lines 164 -169.* |
| **49** | Address the baseline differences between groups, as comparing groups with distinct profiles is a serious concern | We have mentioned it now in the results section. Page 09, lines 264-265. |
| **50** | Examine the differences between groups for specific variables like smokers and RMDQ scores. Clarify how these differences were managed in the analysis. Discuss how the authors addressed the potential bias introduced by one group receiving more treatment, potentially influencing their improvement compared to the control group. Acknowledge this as a potential limitation | A statement is now included as limitation. Discussion section, page 16, lines 439 -443 |
| **51** | Prognostic model: Clarify why the prognostic model was not explicitly stated as the aim of the study | It is stated as one of the aim now. Background section, lines 119 -122. |
| **52** | While revising your submission, please upload your figure files to the Preflight Analysis and Conversion Engine (PACE) digital diagnostic tool, https://pacev2.apexcovantage.com/. PACE helps ensure that figures meet PLOS requirements | Done accordingly |
|  |  |  |
