# Supplementary material for: Effects of pain education on disability, pain, quality of life, and self-efficacy in chronic low back pain: A randomized controlled trial
Source: PLoS One. 2024 May 28;19(5):e0294302. doi: 10.1371/journal.pone.0294302 (PMC11132453; doi:10.1371/journal.pone.0294302)
Supplement: Attachment — Submitted filename: Response to reviewers.doc [file pone.0294302.s005.doc]

April 2024, Ajman, UAE

To: PLOS ONE

**Subject:** Re-Submission of revision required PONE-D-23-35122R1

Effects of pain education on disability, pain, quality of life, and self-efficacy in chronic low back pain: A randomized controlled trial

Dear Editor,

Thank you for your time, consideration, and the suggested review comments.

Please find the “minor revisions” amended in the ‘Revised Manuscript with Track changes’ version.

| S.no | Editors comments | Authors response |
| --- | --- | --- |
| 1. | Sample size calculation; indicate the statistical testing method used for the sample size calculation. Perhaps it is the t-test | Thank you for the suggestion, we have amended the changes accordingly. Page 5, lines 52-53, under Sample size calculation section. |
| 2. | Data analysis section:   a. Typographical error: Student’s t-test b. State the underlying covariance structure used in the linear mixed model and criteria for selecting it. | Thank you, done accordingly.  Page 8: Data analysis section, lines 218-219, lines 223 – 225. |
| 3. | In addition to the mean age, include the corresponding standard deviation, throughout the manuscript. | Thank you, changes amended accordingly.  Results → Baseline characteristics section, page 9, lines 255-256. |
| 4. | Table-1: In the statistical methods section, state & describe the use of all statistical methods.   a. Indicate the statistical testing method used for comparing categorical data. b. State and describe the use of logistic regression modelling | Thank you, please find the changes under Data analysis section, page 7 & 8, lines 217-219.  Page 8, lines 236-242. |
| 5. | To assist in the review process, add line numbering to the document | Done accordingly. |

**Corresponding author/guarantor**

***Corresponding Author:**

Ramprasad Muthukrishnan

College of Health Sciences, Gulf Medical University, Ajman, UAE

Email address: [mrp@gmu.ac.ae](mailto:mrp@gmu.ac.ae)
